# Cognitive Complaints and Their Impact on Daily Life in Patients with Degenerative Cerebellar Disorders

**DOI:** 10.1007/s12311-023-01607-4

**Published:** 2023-10-02

**Authors:** Stacha F.I. Reumers, Dennis J.L.G. Schutter, Roderick P.P.W.M. Maas, Frank-Erik de Leeuw, Roy P.C. Kessels, Bart P.C. van de Warrenburg

**Affiliations:** 1 Department of Neurology, Radboud University Medical Center, Donders Institute for Brain, Cognition and Behaviour, Nijmegen, the Netherlands; 2 Department of Experimental Psychology, Helmholtz Institute, Utrecht University, Utrecht, The Netherlands; 3 Donders Institute for Brain, Cognition and Behaviour, Radboud University, Nijmegen, The Netherlands; 4 Department of Medical Psychology and Radboudumc Alzheimer Center, Radboud University Medical Center, Nijmegen, the Netherlands; 5 Vincent van Gogh Institute for Psychiatry, Venray, The Netherlands

**Keywords:** Ataxia, Cognition, Affect, Disease Burden, Questionnaires, Roy P.C. Kessels and Bart P.C. van de Warrenburg contributed equally to this work.

## Abstract

Cognitive and affective sequelae of cerebellar disease are receiving increased attention, but their actual rate of occurrence remains unclear. Complaints may have a significant impact on patients, affecting social behavior and psychological well-being. This study aims to explore the extent of subjective cognitive and affective symptoms in patients with degenerative ataxias in the Netherlands. An explorative study was set up in a heterogeneous group of degenerative ataxia patients. Self-reported cognition was evaluated in terms of executive functioning and affect (Dysexecutive Questionnaire/DEX), and memory/attention (Cognitive Failures Questionnaire/CFQ). The Daily Living Questionnaire (DLQ) was administered to quantify the impact on daily life. Furthermore, informants completed questionnaires to obtain insight into patients’ self-awareness and social cognition (Observable Social Cognition Rating Scale/OSCARS). This study shows that subjective complaints in the domains of (1) executive functioning and/or (2) memory and attention were reported by 29% of all patients (*n* = 24/84). In addition, more difficulties in daily life in terms of language/comprehension and community/participation were reported, and this was more common for patients with cognitive complaints than those without. Discrepancies between patients and informants about executive functioning were present in both directions. Deficits in social cognition were not identified at the group level, but more social-cognitive problems were observed in patients with more executive problems rated by informants. Taken together, our findings indicate that cognitive complaints are common in patients with degenerative cerebellar disorders and have an impact on daily life functioning. These results may help to increase awareness of cognitive symptoms and their impact in patients with cerebellar ataxia, their significant others, and professional caregivers.

**Supplementary Information:**

The online version contains supplementary material available at 10.1007/s12311-023-01607-4.

## Introduction

Cognitive and affective sequelae of cerebellar disease are receiving increased attention, but their prevalence and impact are still unclear. In addition to the well-documented motor symptoms associated with cerebellar ataxia, cognitive deficits may occur in a wide range of cerebellar disorders, including degenerative ataxias and cerebellar strokes [1]. Symptoms typically include impairments in executive function, visuospatial cognition, affect regulation, and linguistic processing, described as the cerebellar cognitive affective syndrome (CCAS) [2]. The affective component of CCAS mainly includes depressive symptoms, anxiety, and behavioral difficulties [3]. There is a close, bidirectional connection between affect and cognition, where affect can have both (social) cognitive and behavioral consequences [4].

In recent years, several studies on CCAS in neurological patients have been published, but its actual prevalence to date has not been systematically investigated [5–9]. Cognitive performance has previously been studied in relatively small samples of the most common ataxia types [10]. In addition, the vast majority of previous studies has not looked at the prevalence of deficits in the domain of social cognition. In fact, impairments in social cognition are among one of the reported core domains affected in CCAS, with the most prominent deficits in mentalizing (“theory of mind”) and emotion recognition [11–15]. Cognitive complaints may have an impact on behavior and psychological well-being, reducing patients’ quality of life [16]. This might even lead to a larger burden for patients than the motor symptoms, although empirical evidence on the impact of these complaints on daily life, e.g., daily activities and participation, is limited [17]. Determining the prevalence of CCAS is challenging, as its symptoms are not always systematically assessed in clinical practice. Consequently, cognitive and affective symptoms may be underestimated, also because easily applicable instruments are lacking. A brief screening tool (i.e., the CCAS Scale) has recently been developed and translated into several languages, but not all translations have been validated yet or implemented in routine clinical practice [18]. A first step towards establishing the prevalence of CCAS is to gain a more extensive insight into subjectively reported cognitive and affective difficulties in individuals with cerebellar disorders, to better understand patient’s complaints and burden.

This study aims to explore the extent of cognitive and affective symptoms present in a heterogeneous sample of cerebellar patients with degenerative ataxia, with use of self-report and informant-based questionnaires focusing on executive functions, memory, attention, and social cognition. Furthermore, we examined what the impact of these symptoms was on patients’ daily functioning.

## Methods

### Participants and Design

This study was performed in patients with degenerative cerebellar disorders from the Netherlands. Participants were enrolled by the Dutch ataxia patient association (www.ataxie.nl), of which the members are mostly patients with genetic or degenerative cerebellar ataxia. Recruitment was facilitated through announcements on their website, social media, and newsletters. The patient association has about 500 members and we expected an interest to participate of approximately 150 members. To avoid potential selection bias in the recruitment of participants, a general call for participation was used, without mentioning that the study was about cognition. Eligible participants were adult patients with a diagnosis of a degenerative cerebellar ataxia. Information on diagnosis was asked by questions regarding clinical, genetic, and neuroimaging information (e.g., whether patients know that a scan was made that showed “shrinking” of the cerebellum). This information was reviewed by an experienced neurologist (BvdW) and persons were included if this evaluation about diagnosis was sufficiently certain. Exclusion criteria were other neurological disorders or lesions that the patient considered to affect cognitive functioning, such as an established dementia syndrome or stroke. Persons with a diagnosis of depression were included in this study, although depressive symptoms are related to cognitive complaints [19]. However, we aimed to describe cognitive complaints of the entire group of degenerative ataxias, in which depressive symptoms are common and are also an intrinsic component of CCAS [20]. Informants designated by patients were asked to fill in questionnaires about the patient. They were either close family members or friends who knew the patient well. This study was approved by the medical ethics committee (CMO Arnhem-Nijmegen) and all participants provided written informed consent.

### Measures

Standardized questionnaires were used to obtain information about cognition and impact on daily life. Data were gathered digitally; all participants received a link by e-mail to securely fill in the questionnaires at any convenient time. Data were pseudonymized and collected in a Castor EDC database.

*The DysEXecutive questionnaire (DEX)* is a 20-item rating scale evaluating everyday executive functioning on the domains of Affect, Behavior, and Cognition [21]. Both the self-report and informant version were administered. Items were rated on a 5-point Likert scale and the total score ranges from 0 to 80 points. Scores >28 points were an indication for dysexecutive functioning [22]. Also, discrepancy scores were calculated (ranging from −80 to +80 points). Scores in the negative direction indicate that the informant acknowledged more problems than the patient, suggesting impaired self-awareness.

*The Cognitive Failures Questionnaire (CFQ)* is a measure of subjective cognitive function focusing on memory and attention [23]. The questionnaire was filled in by the patient and consists of 25 items regarding the frequency of everyday cognitive failures. In addition, it contains four questions regarding the impact of these failures on daily life. All items were scored on a 5-point Likert scale and the total score ranges from 0 to 100 points. Scores ≥ 43 points were an indication for impaired cognitive function in this domain [24].

*The Daily Living Questionnaire (DLQ), part 1* was used to gain insight into the impact of the complaints on daily life, as it detects difficulties in activities and participation associated with cognitive deficits [25]. This was focused on four domains: Household Tasks, Activities Involving Language/Comprehension, Community/Participation, and Complex Tasks. Patients filled in the questionnaire, which consists of 28 items scored on a 4-point Likert scale. The total score ranges from 28 to 112 points.

*The Observable Social Cognition Rating Scale (OSCARS)* measures social cognition and was administered as an informant-based questionnaire [26]. It consists of eight items on the following domains: Emotion Perception, Attributional Style, Jumping to Conclusions, Cognitive Rigidity, Theory of Mind, and Empathy. Items were rated on a 7-point Likert scale, with total scores ranging from 8 to 56 points.

For all questionnaires, higher scores indicate more problems with cognitive functions or a higher (negative) impact on daily life. Because the DLQ and OSCARS were not available in Dutch language, these questionnaires were translated by three investigators using forward and backward translation (D.S., R.K., S.R., Supplement 1-2). The DEX and CFQ were validated Dutch versions. Scores from the questionnaires were compared with internationally available normative values, and cutoff values for the DEX and CFQ were used to group participants [22, 24, 25, 27–29]. For the DLQ and OSCARS, no cutoff values were available. In addition to the questionnaires, questions about age, sex, education, diagnosis, and symptoms were recorded. Questions about (neurotropic) drug use were asked, including the use of anticholinergics/benzodiazepines. Comorbidities were also asked for, including diagnoses of depression, attention-deficit hyperactivity disorder, autism spectrum disorder, stroke, and other neurological disorders.

### Statistical Analysis

Descriptive statistics were used to determine the rate of occurrence of cognitive and affective complaints. As questionnaire outcomes were not normally distributed, non-parametric tests were used and medians with interquartile ranges (IQR) were reported. One-sample Wilcoxon signed-rank tests were performed to compare the scores of our sample with normative values from the literature. Mann-Whitney *U* tests were used to compare scores between groups with and without cognitive complaints in our sample. The false discovery rate (FDR) approach was applied to correct for multiple comparisons and FDR-adjusted *p*-values were calculated. Spearman’s correlation coefficients were performed to assess associations between variables. All analyses were performed in SPSS Statistics 27.0 (SPSS, Inc., Chicago, IL, USA) and statistical significance was set at 0.05 (two-tailed) for all tests.

Additional analyses were performed to explore the influence of potentially misleading items in the CFQ; this concerns items 5 and 24 (“Do you bump into people?” and “Do you drop things?’). These items may be affected more by motor than cognitive deficits, but were maintained in the questionnaires to be able to compare the outcomes with normative values.

## Results

### Demographic and Clinical Characteristics of Participants

To the general call to participate in this study, 94 persons responded and 87 ultimately completed the questionnaires. Three persons were excluded from the study because of an unclear diagnosis. Out of the 84 patients, 24 (29%) had a “pure” (only cerebellum affected) ataxia and 51 (61%) had “complex” cerebellar ataxia (with additional neurological features). For the remaining nine persons, this was unknown. All characteristics are provided in Table 1. Information obtained by informants was available for 65 patients; the remaining patients did not ask an informant to participate, or the informants refused to fill in the questionnaires. Informants were mostly partners of the patients (*n* = 35, 54%), other informants were first-degree relatives (*n* = 16, 25%) and friends (*n* = 9, 14%). Of five informants, the relationship was unknown (8%).

**Table 1:** Demographic and clinical characteristics of all patients

	All patients (*n* = 84)
Men–women, *n*	37–47
Age, y	58.1 ± 12.4 (28–86)
Age of onset, y	44.0 ± 16.9 (0–80)
Disease duration, y	14.1 ± 12.5 (1–72)
Diagnosis	
-Inherited	69 (82%)
-Sporadic	7 (8.5%)
-Acquired	1 (1%)
-Unknown	7 (8.5%)
Disease stage (self-reported) [47]	
-Stage 0 (no gait difficulties)	10 (12%)
-Stage 1 (onset gait difficulties)	29 (34.5%)
-Stage 2 (loss independent gait)	37 (44%)
-Stage 3 (confinement to wheelchair)	8 (9.5%)
Any comorbidity	28 (33%)
Drug use	51 (61%)
Neurotropic drug use	30 (36%)
-Use of anticholinergics/benzodiazepines	8 (10%)
Education level [48]	5.6 ± 1.1 (1–7)
Subjective cognitive complaints	24 (29%)
-Executive functioning	14 (17%)
-Memory and attention	19 (23%)

Continuous variables are presented as means ± SDs (ranges), ordinal variables as frequencies

One-third of patients had current comorbidities; seven had a diagnosis of depression, three had neuropathy, one had autism spectrum disorder, and one had restless legs syndrome. Furthermore, two persons had a transient ischemic attack (TIA), and one person had a benign brain tumor. The following classes of drugs were taken by the patients: antidepressants (*n* = 9), antiepileptics (*n* = 8), anticholinergics (*n* = 6), benzodiazepines (*n* = 5), spasmolytics (*n* = 4), dopamine agonists (*n* = 3), alpha blockers (*n* = 2), opiates (*n* = 2), acetyl leucine (*n* = 2), riluzole (*n* = 1), antipsychotics (*n* = 1), and sympathomimetics (*n* = 1).

### Subjective Complaints

Of all patients, 24 persons (29%) reported subjective cognitive complaints. Of those, 14 persons reported complaints of executive functioning (17%, DEX score > 28), and 19 persons of memory/attention (23%, CFQ score ≥ 43); nine (11%) reported complaints in both domains. Regarding the characteristics listed in Table 1, persons with and without cognitive complaints were similar except for the prevalence of comorbidities, which were significantly more present in the group of persons with cognitive complaints than in the group without (50% vs. 27%, *p* = 0.04). Complaints regarding memory/attention were positively correlated with a depression (Somers’ *d* = .23, *p* = .032), but not with any other comorbidities. Age had a significant negative correlation with complaints in the domains of memory/attention (*ρ* = −.225, *p* = .039), but not with executive functioning (*ρ* = −.063, *p* = .569). Age of onset was negatively associated with complaints in both the domains of memory/attention (*ρ* = −.310, *p* = .004) and executive functioning (*ρ* = −.223, *p* = .042). No associations were found between subjective cognitive complaints and sex, education level, disease stage, disease duration, neurotropic drug use, or groups of diagnosis (all *p* values > .05). Median scores of the DEX and CFQ in all patients were not significantly different from normative values in healthy individuals. Patients with cognitive complaints scored significantly higher than the group without complaints on all subscales of the DEX: Affect, Behavior, and Cognition (all *p* values = .001). Results of the DEX and CFQ are listed in Table 2.

**Table 2:** Outcomes of the Dysexecutive Questionnaire (DEX) and Cognitive Failures Questionnaire (CFQ)

	Group with cognitive complaints (*n* = 24)	Group without cognitive complaints (*n* = 60)	All patients (*n* = 84)	Normative values
DEX patient (range 0–80)	30 (19–38)*	14 (10–19)	16.5 (13–23)	17.10 (12–23)
-Affect (range 0–12)	5.5 (3–7)*	2 (1–4)	3 (2–5)	
-Behavior (range 0–32)	12 (7–16)*	5.5 (3–9)	6.5 (4–11)	
Cognition (range 0–20)	8 (6–9)*	3 (1–5)	4 (2–7)	
CFQ (range 0–100)	45 (43–51)*	24.5 (18–31)	29.5 (21–41)	31.8 (24–39)

Variables are presented as medians with interquartile ranges (IQR)

*Significantly different from the group without complaints

An explorative analysis on the CFQ without two items (5 and 24, see “Methods”) did not affect the classification of participants into groups. Total median scores were slightly lower for the group with complaints (42.5), group without complaints (22), and all patients (26). The differences between the groups with and without cognitive complaints remained statistically significant (*p* < .01).

### Discrepancies Between Patients and Informants

**Fig. 1:**
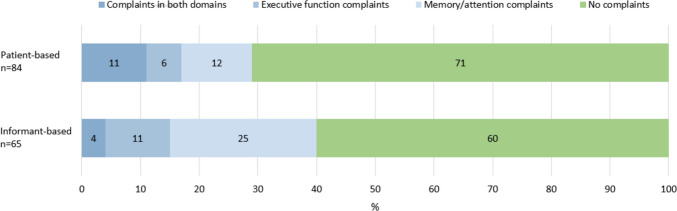
Relative distribution of participants based on patients’ (upper bar) or informants’ (lower bar) scores on the Dysexecutive questionnaire. Percentages of patients with (1) complaints in both domains, (2) only executive function complaints, (3) only memory/attention complaints, and (4) no complaints are depicted

Discrepancies were noticed when looking at DEX scores provided by the patients and informants; this is depicted in Fig. 1. In total, 29% of patients reported cognitive complaints, which was higher (40%) when considering scores based on informants (*n* = 65). Whether taking the patients’ or informants’ point of view as reference influences the numbers of patients with and without cognitive complaints. There were no differences in discrepancies between male patients and female informants, as compared to female patients and male informants.

**Fig. 2:**
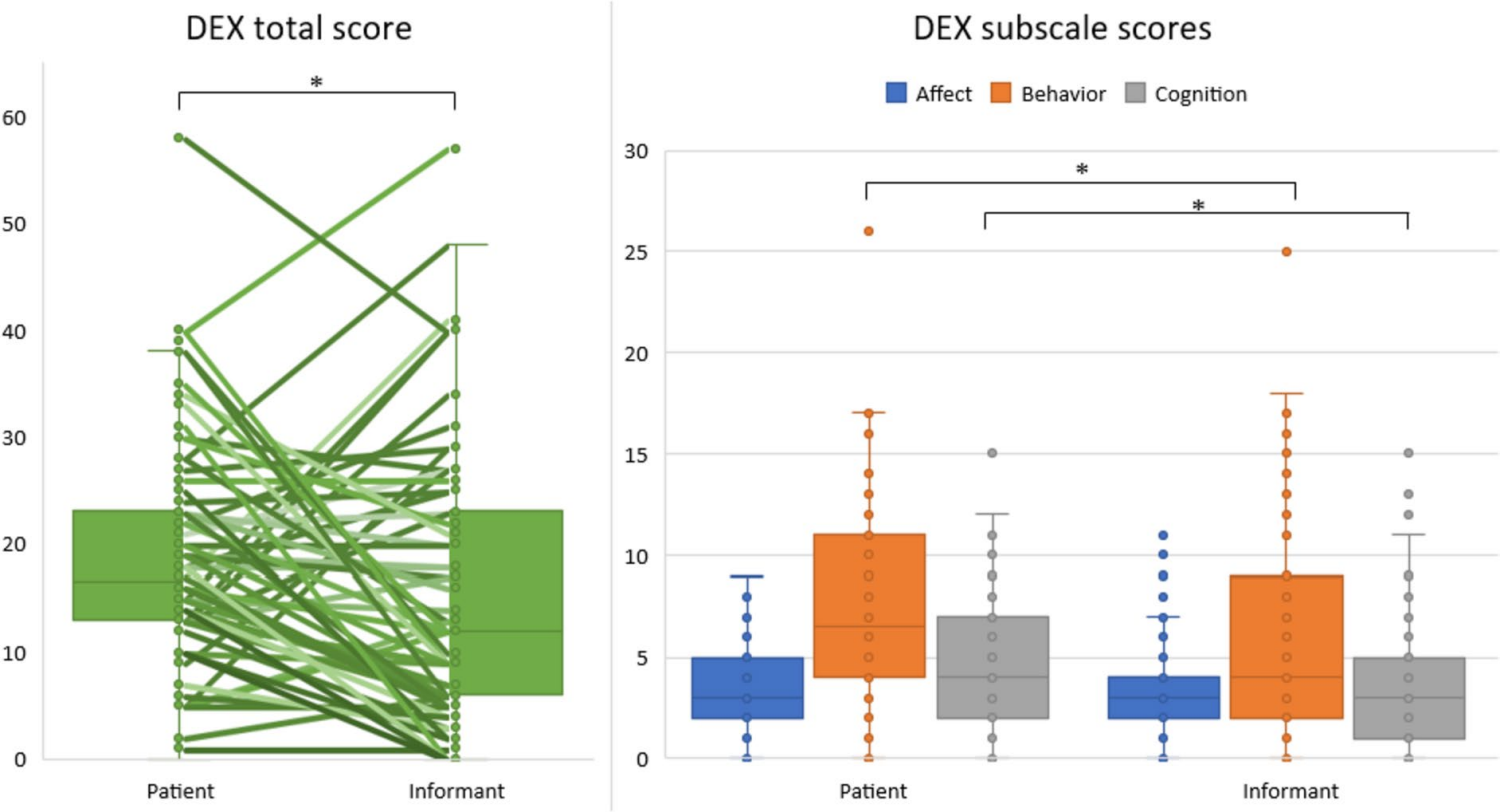
Outcomes of the Dysexecutive questionnaire between patients and informants. Boxplots are depicted with medians and interquartile ranges. Additionally, discrepancies are shown for the total score, in which every line represents one participant. *Significant differences between patients and informants (*p* < .05)

Discrepancies in the DEX scores were calculated for insight into self-awareness and yielded a median difference between patients and informants of 4 points (range −27 to +32 points). When comparing patients’ DEX scores to informants’ scores, the self-reported problems were on average significantly higher for the total score (*p* = .017), as well as the subscales Behavior (*p* = .023) and Cognition (*p* = .020), but no differences were found for the subscale Affect (*p* = .219). This is visualized in Fig. 2.

### Social Cognition

Social cognition was described using the OSCARS questionnaire filled in by informants (*n* = 63); two informants did not complete the questionnaire. Total scores were comparable to the available normative values since these were not statistically different [27]. No significant difference was found between the participants with and without cognitive complaints. All outcomes are listed in Table 3. Positive correlations were found between the patients’ DEX scores and OSCARS total score (*ρ* = .352, *p* = .005). A positive correlation was also observed between the DEX and the OSCARS domains of Cognitive Rigidity (*ρ* = .303, *p* = .015) and Theory of Mind (*ρ* = .353, *p* = .004), but not for the other domains. Informants’ DEX scores for all subscales and the total score were positively correlated with all OSCARS scores (*ρ* = .458–.856, *p* < .001). Also, positive associations were found (*ρ* = .331–.488, *p* < 0.01) between all OSCARS items and patients with a DEX discrepancy score below −10. This implies that more social-cognitive problems were observed in those patients in whom more daily executive problems were reported by the informant than acknowledged by the patient, since discrepancy scores below −10 indicate that informants reported notably more difficulties than the patients themselves. No significant correlations were found between OSCARS scores and the CFQ.

**Table 3:** Outcomes of the Observable Social Cognition Rating Scale (OSCARS)

	Group with cognitive complaints (*n* = 15)	Group without cognitive complaints (*n* = 48)	All patients (*n* = 63)	Normative value
OSCARS (range 8–56)	15 (9–25)	11 (9–15.8)	11 (9–17)	12.1 (9–15)
-Emotion perception	1 (1–3)	1 (1–2)	1 (1–2)	
-Attributional style	1 (1–2)	1 (1–1)	1 (1–2)	
-Jumping to conclusions	1.5 (1–4.8)	1 (1–2)	1 (1–3)	
-Cognitive rigidity	1.5 (1–3.4)	1 (1–1.5)	1 (1–2)	
-Theory of mind	1.7 (1.3–3)	1.3 (1–2)	1.3 (1–2.3)	

Variables are presented as medians with interquartile ranges (IQR)

### Impact on Daily Life

**Fig. 3:**
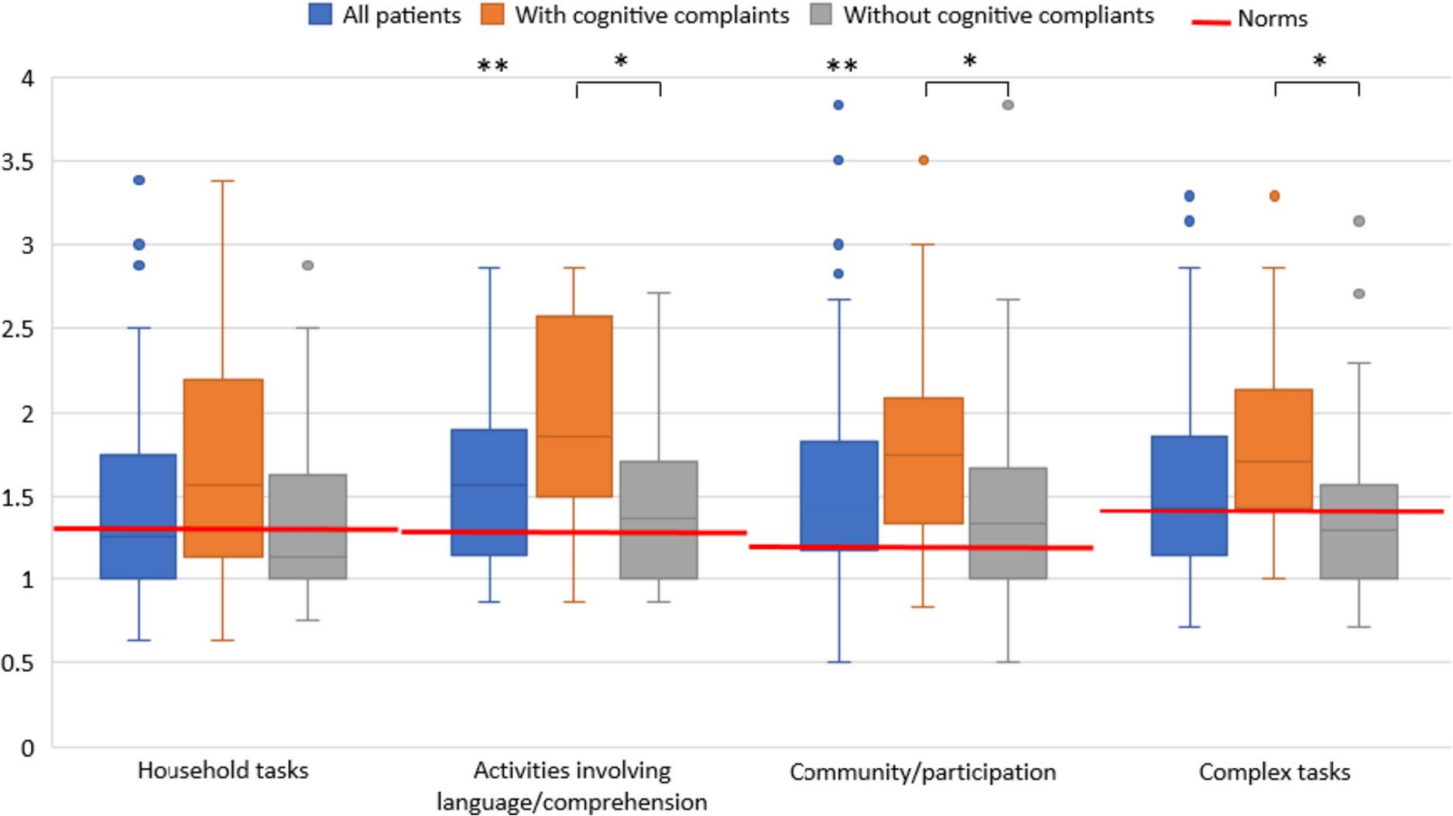
Outcomes of the Daily Living Questionnaire in all patients (blue), the group with cognitive complaints (orange), and the group without cognitive complaints (gray) compared with normative values from the literature (red lines). *Significant differences between groups (*p* < .05). **Significantly different from normative values (*p* < .001)

The relation between subjective cognitive complaints and the impact on daily life was evaluated. Compared with normative values, the entire study sample scored significantly higher on the DLQ subscales for Activities Involving Language/Comprehension (*p* < .001) and Community/Participation (*p* < .001), but not for the subscales for Household- and Complex Tasks. Details on the scores are shown in Table 4. The group with cognitive complaints scored significantly higher on the DLQ subscales for Activities Involving Language/Comprehension (*p* = .001), Community/Participation (*p* = .001), and Complex Tasks (*p* = .017), but not on the subscale for Household Tasks (*p* = .092). This is shown in Fig. 3. Furthermore, scores on the four CFQ questions regarding impact on daily life were significantly higher in the group with cognitive complaints (*p* < .001) than in the group without, indicating that patients reported more hindrance, worries, and annoyance as consequences of the complaints. Spearman’s tests revealed positive correlations between all DLQ subscales and the CFQ and DEX (*ρ* = .274–.398, *p* < .01). Also, a correlation was found between the DEX Affect items and the subscale for Activities Involving Language/Comprehension of the DLQ (*ρ* = .352, *p* = .001).

**Table 4:** Questionnaire outcomes of all patients

	Group with cognitive complaints (*n* = 24)	Group without cognitive complaints (*n* = 60)	All patients (*n* = 84)	Normative values
DLQ				
-Household Tasks	1.56 (1.1–2.3)	1.13 (1–1.6)	1.25 (1–1.8)	1.27 (1–1.5)
-Language/Comprehension	1.86 (1.4–2.6)*	1.36 (1–1.7)	1.57 (1.1–2)**	1.26 (1–1.5)
-Community/Participation	1.75 (1.3–2.3)*	1.33 (1–1.7)	1.42 (1.2–1.8)**	1.19 (1–1.4)
-Complex Tasks	1.71 (1.4–2.1)*	1.29 (1–1.6)	1.43 (1.1–1.9)	1.41 (1–1.7)
CFQ Increase	3 (2–4)*	2 (1–2)	2 (2–3)	
CFQ Hindrance	3 (3–4)*	2 (2–3)	2.5 (2–3)	
CFQ Worries	3 (3–3.8)*	2 (1–3)	2 (1–3)	
CFQ Annoyance	3 (2–4)*	2 (1–3)	2 (2–3)	

Variables are presented as medians with interquartile ranges (IQR)

*Significantly different from the group without complaints

**Significantly different from normative values (*p* < .001)

## Discussion

The present study describes the extent of cognitive and affective symptoms in a large and heterogeneous group of patients with degenerative cerebellar ataxias. Key findings are that 29% of patients reported subjective cognitive complaints in the domains of (1) memory and attention and/or (2) executive functioning. The entire group of patients taken together did on average not report more cognitive complaints than healthy controls. However, persons with degenerative cerebellar disease experienced significantly more difficulties in daily life regarding activities involving language/comprehension and community/participation, and this was more common for patients with cognitive complaints than without. Discrepancies between patients and informants were observed, with patients reporting on average more problems with their executive functioning than their informants. At the group level, no major deficits in social cognition of the patients were observed by the informants compared to available reference values. However, informants who noted more problems in executive functioning in patients also noted more social cognition-related problems in these persons.

In this study, subjective cognitive complaints occurred in 29% (*n* = 24) of the patients with degenerative cerebellar ataxias. Recently, a review was published in which the prevalence of cognitive symptoms in spinocerebellar ataxias was estimated between 23 and 75% [30]. This broad range was based on a variety of articles that applied different methods of identifying the cognitive sequalae of cerebellar disorders. Some performed extensive neuropsychological testing, while others administered only short cognitive screens, such as the Mini-Mental State Examination (MMSE) or used self-report questionnaires. Our results are restricted to data from patients in the Netherlands only. Two other recent studies showed a high prevalence of cognitive deficits in patients with cerebellar strokes and Friedreich’s ataxia, and concluded that the majority of participants manifested CCAS (59–84%) [31, 32]. However, the diagnosis of CCAS was based on the CCAS scale, an instrument that has not been validated extensively yet and that may have a suboptimal specificity, resulting in a relatively high number of false positive classifications [33].

In this study sample of 84 patients with cerebellar degenerative ataxia, we found that 71% had no complaints. Possibly, patients may have downplayed everyday problems or may be less aware of such problems that have arisen during the slowly progressive disease course. Impaired self-awareness could be suggested based on the pronounced discrepancies between patients and informants in our sample. Some patients rated their executive function performance much better compared with their informants’ ratings, while others scored their performance much worse. However, on average, patients reported more problems than their informants. Patients with cognitive problems may have reduced awareness, as has been observed in patients with Huntington’s (HD) or Parkinson’s disease (PD) [34]. Impaired self-awareness in ataxia patients has been indicated recently in a small study regarding motor symptoms and may also extend to cognitive symptoms [35]. In turn, informants may have either exaggerated or downplayed problems as a coping mechanism. In this case, patients more accurately reflect on their functioning, as has also been observed in patients with multiple sclerosis (MS) [36, 37]. Nevertheless, it is unclear whether measures based on patients’ or informants’ self-report are more valid, since both are subjective, and we did not include objective test performances to correlate with these self-report measures.

Complaints regarding memory and attention had a weak, negative correlation with age. This seems counterintuitive, as more subjective complaints are typically associated with a higher age [38]. However, it may be that older persons may notice less problems because of less demanding engagements in later stages of life and/or, alternatively, accept these as part of the aging process. More cognitive complaints were also weakly correlated to a lower age of onset, but not to disease duration. This could imply that the age at which the cerebellar disorder manifests has an effect on the severity of cognitive dysfunction, but this warrants further study. Previous studies remain inconclusive about whether cognitive dysfunction is associated with age at onset or disease duration. Cognitive complaints were not correlated with disease stage/motor symptoms. This underlines that even when motor symptoms are mild, cognitive deficits may be present. An explorative analysis was performed without two potentially misleading items of the CFQ, but this did not yield divergent outcomes, except for total CFQ scores, which decreased from 29.5 to 26.0 points, reflecting a slight overestimation of scores.

The informant data provided a unique opportunity to report on social cognitive functioning as observed by others than the ataxia patients themselves. This domain is a crucial component of CCAS, but often neglected in empirical studies on cerebellar disorders. Ratings by informants typically correlate better with objective measures of social cognition than ratings by patients, as evidenced from studies in other neurological disorders [39, 40]. On a group level, our results did not differ from normative values and no differences were found between participants with and without self-reported cognitive complaints. The OSCARS has previously been used in patients with schizophrenia, who have substantially higher scores than our cohort [27]. However, we found moderate to strong correlations between OSCARS ratings and informants’ scores on executive functioning, confirming its construct validity.

Cerebellar patients reported more impact on daily life in the domains of activities involving Language/Comprehension and Community/Participation, while previous studies have shown that patients with MS or PD only reported more problems in Community/Participation than healthy controls [25, 41]. Higher-order cognition is needed for convenient participation in a community, such as planning and participating in social activities or hobbies. As the problems in the domain of activities involving Language/Comprehension differed from other comparable disease samples, they may be more specific to people with cerebellar disorders. Examples of difficulties in this domain include reading books, following a conversation, and expressing one’s thoughts. Indeed, there is evidence for a role of the cerebellum in language [42]. Also, language and comprehension require higher-order cognition, such as attention and working memory. Patients with cognitive complaints reported more difficulties with activities involving Language/Comprehension, Community/Participation, and Complex Tasks than patients without cognitive complaints. They also reported more hindrance, worries, and annoyance, implying that their complaints may have a significant impact on daily life. A moderate correlation was found between cognitive complaints regarding affect and difficulties in the domain of language/comprehension. Positive, but weak, correlations were found between the impact on daily life and complaints about executive functioning and memory/attention. This link with daily life is an important aspect to take into account for health professionals in clinical care.

Possible confounders in this study may be related to comorbidities and (neurotropic) drug use. One-third of all patients had comorbidities and this proportion was higher in the group with cognitive complaints (50%). Depression was most often reported as comorbidity and six out of seven persons with a depression also experienced subjective cognitive complaints in this sample. Depressive symptoms are known to be associated with cognitive complaints in general, but also with executive dysfunction and memory/attentional defects [19, 43]. This is in line with our observations, as a positive correlation was found between having a depression and complaints reported on the CFQ. Depressive symptoms can be (1) a primary consequence of a cerebellar disease, since the cerebellum is connected with the affective circuit [44]; (2) a secondary response to motor difficulties; or (3) a combination of both. Neurotropic drugs were used by 36% of patients and could also have affected cognitive function. Benzodiazepines and anticholinergics are specifically associated with worse cognitive performance and were taken by 10% of patients [45]. However, while no correlations were observed between medication use and cognitive complaints in our sample, an association cannot be completely ruled out.

Our study has a number of limitations. The Dutch ataxia patient association has about 500 members, and 94 persons responded to the announcement of this study of whom the majority (93%) filled out the questionnaires. Selection bias has likely occurred, as persons with digital illiteracy probably have not participated in our online questionnaire study. However, we presume selection bias to be limited as a general call for participation was used, without mentioning that questionnaires would be about cognition. This study is further limited as we used subjective self-report measures on cognitive (dys)function and everyday activities, without using an objective measure. Subjective complaints are influenced by psychological well-being and fatigue, which might be confounding [46]. Information about formal diagnoses of depression, schizophrenia, obsessive compulsive disorder, and addiction was not specifically included in this study. However, subjective cognitive complaints in patients with ataxia have rarely been investigated. More knowledge on the cognitive aspects in cerebellar disorders is relevant for clinical practice, to gain awareness for healthcare providers and for patients with complaints to feel acknowledged. This is crucial to obtain optimal patient care. Future research should extend our findings by also performing objective neuropsychological tests, preferably on a larger scale and internationally. Longitudinal studies would be useful to gain insight into the course of the disease and symptoms. This information is needed to develop and assess effective interventions.

## Conclusion

Cognitive complaints are common in patients with degenerative cerebellar ataxias, which are mainly found in the domain of memory and attention, but also in executive functioning (including affect) or both. Cerebellar patients experienced significant difficulties in daily life in terms of activities involving language/comprehension and community/participation, implying that cognitive complaints have an added impact on daily life functioning. Finally, informants who reported more problems with executive functioning in patients also noted more difficulties in social cognitive functioning of these persons. These results may increase acknowledgement, recognition, and awareness of cognitive symptoms and their impact on daily life. This could ultimately lead to better management of patients with cerebellar disorders.

### Supplementary Information

ESM 1: (PDF 176 kb)

ESM 2: (PDF 112 kb)

## Data Availability

The data that support the findings of this study are available upon reasonable request.
