# Supplementary material for: Cognitive Complaints and Their Impact on Daily Life in Patients with Degenerative Cerebellar Disorders
Source: Cerebellum. 2023 Oct 2;23(3):1042–52. doi: 10.1007/s12311-023-01607-4 (PMC11102381; doi:10.1007/s12311-023-01607-4)
Supplement: Supplementary file 1 — (PDF 176 kb) [file 12311_2023_1607_MOESM1_ESM.pdf]

Vragenlijst Dagelijks Leven – NL (Daily Living Questionnaire – R) **versie 1.0**

Naam: **Datum:**

Elke dag voeren we veel taken uit die vragen om mentale inspanning, ook wel cognitie genoemd. In de loop van ons leven ondervinden we soms moeilijkheden met deze mentale inspanning of cognitieve activiteiten. Hieronder volgt een lijst met activiteiten die u wellicht in uw dagelijks leven uitvoert.

Omcirkel het getal dat het beste beschrijft in hoeverre u mentale of cognitieve last ervaart bij het uitvoeren van elk van de onderstaande activiteiten. Als de activiteit niet op u van toepassing is, omdat u bijvoorbeeld deze activiteit nog nooit gedaan hebt, omcirkel dan 'Niet van toepassing (n.v.t.)'.

**Deel 1: Hoeveel moeite heeft u bij het uitvoeren van de volgende taken?**	**GEEN mentale of cognitieve moeite**	**ENIGE mentale of cognitieve moeite**	**VEEL mentale of cognitieve moeite**	**Niet in staat uit te voeren**	**Niet van toepassing (n.v.t.)**
1. Wassen en aankleden in de ochtend	1	2	3	4	9
2. Het vinden van spullen op een volle plank of in een kast	1	2	3	4	9
3. Inrichten van kasten / planken / lades	1	2	3	4	9
4. Voorbereiden en klaarmaken/bereiden van maaltijden	1	2	3	4	9
5. Huishoudelijke taken (de was doen)	1	2	3	4	9
6. Winkelen (kopen wat u nodig heeft, beslissingen nemen, spullen vinden)	1	2	3	4	9
7. Organiseren en plannen van eigen dagelijkse activiteiten en klusjes	1	2	3	4	9
8. Plannen / kiezen welke kleding u gaat dragen	1	2	3	4	9
9. Kranten / tijdschriften lezen	1	2	3	4	9
10. Boeken lezen	1	2	3	4	9
11. Zoeken naar informatie (op internet, in de bibliotheek, etc.)	1	2	3	4	9
12. Het uiten van uw gedachten	1	2	3	4	9
13. Een gesprek volgen	1	2	3	4	9

Vragenlijst Dagelijks Leven – NL (Daily Living Questionnaire – R) **versie 1.0**

Deel 1: Hoeveel moeite heeft u bij het uitvoeren van de volgende taken?	GEEN mentale of cognitieve moeite	ENIGE mentale of cognitieve moeite	VEEL mentale of cognitieve moeite	Niet in staat uit te voeren	Niet van toepassing (n.v.t.)
14. Deelnemen aan groepsgesprekken	1	2	3	4	9
15. Het schrijven van een brief of verslag	1	2	3	4	9
16. Plannen van sociale afspraken met familie, vrienden of voor kinderen	1	2	3	4	9
17. Deelnemen aan sociale activiteiten met anderen	1	2	3	4	9
18. Deelnemen aan recreatieve activiteiten, vrije tijd, hobby's	1	2	3	4	9
19. Een drukke straat oversteken	1	2	3	4	9
20. Autorijden	1	2	3	4	9
21. Geld pinnen bij een automaat	1	2	3	4	9
22. Het invullen van aanvragen en formulieren	1	2	3	4	9
23. Dingen repareren	1	2	3	4	9
24. De weg vinden in onbekende omgevingen	1	2	3	4	9
25. Rekenen/berekeningen maken	1	2	3	4	9
26. Bijhouden en beheren van financiën	1	2	3	4	9
27. Rekeningen betalen	1	2	3	4	9
28. Het volgen van schriftelijke instructies	1	2	3	4	9

© 2011 J. Toglia
Nederlandstalige vertaling en bewerking: Stacha F.I. Reumers, Dirk Bertens, Dennis J.L.G. Schutter, Roy P.C. Kessels.
