# Supplementary material for: Cognitive Complaints and Their Impact on Daily Life in Patients with Degenerative Cerebellar Disorders
Source: Cerebellum. 2023 Oct 2;23(3):1042–52. doi: 10.1007/s12311-023-01607-4 (PMC11102381; doi:10.1007/s12311-023-01607-4)
Supplement: Supplementary file 2 — (PDF 112 kb) [file 12311_2023_1607_MOESM2_ESM.pdf]

Deelnemer ID: Bezoek #:
Deelnemer initialen: Bezoek datum:

OSCARS-NL (Observatie van Sociale Cognitie - Informantvragenlijst) versie 1.0

David Penn, Dennis Combs, David Roberts, Steven Silverstein, & Richard Keefe.

Nederlandstalige vertaling en bewerking: Stacha Reumers, Dennis Schutter & Roy Kessels.

Relatie met deelnemer:

Afnamedatum:

Deze vragen hebben allemaal betrekking op de deelnemer aan het onderzoek. U als naaste kent hem of haar goed, vandaar dat we aan u vragen om deze lijst in te vullen over sociale situaties die u met hem/haar meemaakt. We willen daarom ook uw oordeel weten, en u vragen niet met de deelnemer te overleggen over welk antwoord het meest gepast is.

Wij vragen u op de onderstaande schaal aan te geven of de deelnemer aan het onderzoek problemen heeft in acht domeinen. Beoordeel steeds de ernst van de (mogelijke) problemen van 1 (geen) tot 7 (zeer ernstig). Hogere scores geven dus ernstigere problemen aan.

Beoordeel het gedrag van de persoon in de afgelopen 7 dagen. Let hierbij op ieder contact dat u met haar/hem heeft gehad, en elk contact dat u heeft opgemerkt tussen haar/hem en andere mensen.

Bij ieder afzonderlijk item geven we een algemeen voorbeeld van het gedrag in kwestie, de beoordelingsschaal (1-7), de mate van ernst (geen tot zeer ernstig) en gedragsbeschrijvingen die overeenkomen met de beoordelingen. Beoordeel alle items. Geef uw beoordeling door het nummer op de schaal voor dat item te omcirkelen. U kunt elk getal op de schaal gebruiken.

Niveau van ernst beoordelingsschaal

**1**	**2**	**3**	**4**	**5**	**6**	**7**
**Geen**	**Zeer licht**	**Licht**	**Matig**	**Matig tot ernstig**	**Ernstig**	**Zeer ernstig**

Deelnemer ID: Bezoek #:
Deelnemer initialen: Bezoek datum:

Heeft de persoon moeite in de volgende gebieden?

1. Herkennen van emoties van anderen, vooral negatieve emoties (verdriet, angst en boosheid) op basis van gezichtsuitdrukking, lichaamstaal en/of stemtoon en snelheid?

Bijvoorbeeld: Wanneer hij/zij met iemand praat, kan de persoon niet zeggen of de andere persoon van streek of boos is. Hij/zij lijkt geen idee te hebben hoe andere mensen zich voelen.

1	**Geen**	Kan sterke, matige en subtiele uitingen van emoties herkennen. Hij/zij kan worden gezien als 'sociaal opmerkzaam'.	
2	**Zeer licht**		**Informant**
3	**Licht**	Herkent gemakkelijk sterke en duidelijke uitingen van emoties, zoals huilen, boos geschreeuw of opgetogen lachen. Herkent wellicht niet minder duidelijk geuite emoties. Hij/zij herkent subtiele uitdrukkingen echter niet, zoals teleurstelling wat alleen uitgedrukt wordt met een zucht of een lichte mond beweging.	**1 2 3 4 5 6 7**
4	**Matig**		
5	**Matig tot ernstig**	Herkent de meeste sterke en duidelijke uitingen. Herkent geen minder duidelijke of subtiele emotionele uitingen.	
6	**Ernstig**		
7	**Zeer ernstig**	Herkent geen sterke, matige en subtiele emotionele uitingen. De persoon moet worden verteld welke emotie wordt geuit. ("Ik ben heel boos.")	

2. Het interpreteren van sociale interacties op een kwaadwillende, vijandige manier?

Bijvoorbeeld: De persoon meent dat anderen de bedoeling hebben hem/haar leed toe te brengen, vooral in dubbelzinnige (onduidelijke) situaties. Hij/zij loopt bijvoorbeeld langs een paar mensen die lachen en denkt dan dat ze om hem/haar lachen. Of de persoon kan een persoonlijk voorwerp niet vinden en denkt dat iemand anders het heeft gepakt (let hierbij ook erop hoe vaak dit voorkomt)

1	**Geen**	De persoon geeft anderen niet meteen de schuld of denkt niet dat ze slechte bedoelingen hebben met hem/haar. Hij/zij zal doorgaans vinden dat andere factoren, zoals iets in de omgeving, de uitkomst hebben veroorzaakt (bijv. anderen lachen om een grap, niet om hem/haar).	
2	**Zeer licht**		**Informant**
3	**Licht**	Heeft soms het gevoel dat anderen van plan zijn hem/haar pijn te doen, wat van voorbijgaande aard of korte duur kan zijn.	**1 2 3 4 5 6 7**
4	**Matig**		
5	**Matig tot ernstig**	Interpreteert het gedrag van anderen vaak als slecht bedoeld. Beschuldigt naasten soms van kwade wil.	
6	**Ernstig**		
7	**Zeer ernstig**	Heeft uitgebreide overtuigingen van kwade wil, bij zowel naasten als onbekenden. Deze overtuigingen zijn over het algemeen ongegrond.	

Deelnemer ID: Bezoek #:
Deelnemer initialen: Bezoek datum:

3. Neemt beslissingen snel (dat wil zeggen trekt overhaaste conclusies) zonder andere mogelijkheden te overwegen?

Bijvoorbeeld: De persoon probeert u te bellen en u neemt niet op. Hij/zij gelooft meteen dat u niet opnam omdat u boos op hem/haar bent. (Vraag naar de frequentie)

**1**	**Geen**	Neemt geen overhaaste beslissingen; is bedachtzaam en weloverwogen. De persoon neemt andere informatie in ogenschouw en neemt de tijd om de voor- en nadelen zorgvuldig af te wegen alvorens een beslissing te nemen.	
**2**	**Zeer licht**		**Informant**
**3**	**Licht**	Gebruikt soms alleen direct beschikbare informatie om beslissingen te nemen. De persoon neemt soms beslissingen met behulp van aanvullende informatie en kan de voor- en nadelen afwegen indien gemotiveerd.	**1 2 3 4 5 6 7**
**4**	**Matig**		
**5**	**Matig tot ernstig**	Gebruikt vaak alleen direct beschikbare informatie om beslissingen te nemen. De persoon moet worden gevraagd of verteld om andere mogelijkheden te onderzoeken en de tijd te nemen voor het maken van beslissingen.	
**6**	**Ernstig**		
**7**	**Zeer ernstig**	Gebruikt vaak alleen de meest direct beschikbare informatie om beslissingen te nemen. De persoon zoekt geen aanvullende informatie en lijkt bijna zonder na te denken meteen te oordelen. Lijkt irrationeel.	

4. Flexibel zijn in het interpreteren van sociale situaties?

Bijvoorbeeld: De persoon wacht op iemand in een restaurant en de persoon is 20 minuten te laat. Hij/zij concludeert dat de ander hem/haar niet wil ontmoeten, terwijl deze persoon in werkelijkheid vast zit in het verkeer of een lekke band heeft gekregen. Hij/zij denkt niet na over andere mogelijkheden waarom de persoon te laat is en blijft bij één mening.

**1**	**Geen**	Bij het overdenken van het gedrag van iemand anders, kan hij/zij meerdere redenen aandragen waarom de andere handelde zoals hij/zij deed.	
**2**	**Zeer licht**		**Informant**
**3**	**Licht**	Heeft wat moeite met het bedenken van verklaringen, maar kan dit wel als hij/zij gemotiveerd is.	**1 2 3 4 5 6 7**
**4**	**Matig**		
**5**	**Matig tot ernstig**	Heeft moeite om meerdere mogelijke verklaringen te bedenken voor andermans sociaal gedrag; moet worden gevraagd om met verschillende verklaringen te komen over het gedrag van de ander.	
**6**	**Ernstig**		
**7**	**Zeer ernstig**	Begrijpt niet dat meer dan één interpretatie van een gebeurtenis mogelijk is en kan geen alternatieve verklaringen bedenken.	

Deelnemer ID:	Bezoek #:
Deelnemer initialen:	Bezoek datum:

5. In staat zijn om onjuiste interpretaties van sociale interacties aan te passen of te corrigeren?

Bijvoorbeeld: De persoon ziet twee mensen fluisteren en denkt dat ze het over hem/haar hebben. Deze interpretatie wordt volgehouden, zelfs wanneer de persoon wordt verteld dat een van de mensen iets persoonlijks over zichzelf deelde.

1	**Geen**	Is in staat bewijs te zoeken voor en tegen een bepaalde interpretatie over iemand anders, en dit af te wegen.	
2	**Zeer licht**		**Informant**
3	**Licht**	De persoon zal aanwijzingen die de interpretatie van hem/haar tegenspreekt in overweging nemen, maar blijft alsnog bij zijn/haar onjuiste interpretatie.	**1 2 3 4 5 6 7**
4	**Matig**		
5	**Matig tot ernstig**	De persoon luistert vaak niet naar feiten die zijn/haar interpretatie tegenspreken, of kan sterk tegen deze feiten pleiten, en blijft dan bij de onjuiste interpretatie.	
6	**Ernstig**		
7	**Zeer ernstig**	De persoon weigert feiten die zijn/haar interpretatie tegenspreken in overweging te nemen. Het is onmogelijk om de persoon op andere gedachten te brengen, zelfs als de interpretatie duidelijk niet klopt.	

6. Het begrijpen van subtiele grappen, sarcasme en beledigingen in een gesprek?

Bijvoorbeeld: Iemand zegt tijdens een maaltijd op een sarcastische toon dat dit het beste eten is dat hij/zij ooit heeft gehad en de persoon beseft niet dat dit een belediging en/of sarcasme is.

1	**Geen**	De persoon begrijpt subtiele grappen, beledigingen of sarcasme.	
2	**Zeer licht**		**Informant**
3	**Licht**	De persoon begrijpt subtiele grappen of beledigingen die gemaakt worden door anderen soms niet (bijv. “O ja, ik werk graag 15 uur per dag!”). Het lijkt langer te duren om de betekenis van grappen en sarcasme te begrijpen.	**1 2 3 4 5 6 7**
4	**Matig**		
5	**Matig tot ernstig**	De persoon begrijpt subtiele grappen, sarcasme of beledigingen vaak niet en moet dan worden verteld wat de ander bedoelt.	
6	**Ernstig**		
7	**Zeer ernstig**	De persoon heeft geen notie van nuances. Uitspraken moeten concreet en direct zijn om begrepen te worden (bijv. slapstickhumor).	

7. Zaken bekijken vanuit het perspectief van anderen (dat wil zeggen zichzelf in andermans schoenen kunnen verplaatsen)?

Bijvoorbeeld: De persoon kan niet begrijpen waarom iemand in een bepaalde situatie van streek is of zich boos voelt. Of voelt zich niet ontroerd als hij/zij naar een verdrietige film kijkt.

1	**Geen**	De persoon kan worden omschreven als empathisch. Bij het kijken naar een verdrietige of vrolijke film (of het lezen van een verdrietig of vrolijk boek), kan hij/zij erdoor geraakt worden.	
2	**Zeer licht**		**Informant**
3	**Licht**	Vraagt zelden spontaan naar de gedachten of gevoelens van anderen (bijv. "Vind je dat leuk?" of "Ben je in de war?"), maar begrijpt het wanneer mensen dergelijke uitspraken doen.	**1 2 3 4 5 6 7**
4	**Matig**		
5	**Matig tot ernstig**	Reageert alleen empathisch wanneer anderen sterke emoties uiten. Vraagt niet naar de meningen of ervaringen van anderen of reageert hier niet op. Desgevraagd kan hij/zij moeite hebben om zich voor te stellen wat anderen zouden kunnen denken.	
6	**Ernstig**		
7	**Zeer ernstig**	Niet in staat om goed te beoordelen wat anderen zouden kunnen denken of voelen, behalve in de meest extreme situaties (zoals verdriet voelen na het overlijden van een geliefde).	

8. Het begrijpen van subtiele sociale signalen, hints en indirecte verzoeken (een voorbeeld van een indirect verzoek is als uw zoon/dochter speelgoed wilt en dat niet direct zegt, maar wel opmerkt hoe mooi het is.)

Bijvoorbeeld: U probeert een boek te lezen of tv te kijken en de persoon blijft tegen u praten, ook al geeft u met subtiele hints/signalen aan dat u op dat moment niet geïnteresseerd bent om met hem/haar te praten (bijv. korte antwoorden geven; geen oogcontact maken).

1	**Geen**	De persoon pikt gemakkelijk sociale signalen en/of indirecte verzoeken op. Als u het bijvoorbeeld druk hebt en hij/zij begint tegen u te praten, merkt hij/zij snel op dat u op dat moment niet aanspreekbaar bent.	
2	**Zeer licht**		**Informant**
3	**Licht**	De persoon pikt in eerste instantie geen subtiele sociale signalen op, maar doet dat wel na een minuut of twee. Het duurt langer om subtiele signalen en hints op te vangen.	**1 2 3 4 5 6 7**
4	**Matig**		
5	**Matig tot ernstig**	De persoon pikt geen sociale signalen op; moet een aantal duidelijke signalen krijgen om de boodschap over te laten komen (bijv. zich afwenden wanneer iemand met hem/haar praat).	
6	**Ernstig**		
7	**Zeer ernstig**	De persoon pikt geen sociale signalen op en deze moeten op een duidelijke manier worden verteld. Of de persoon vangt geen subtiele hints of indirecte verzoeken op.	
